# A Photoelectrochemical Biosensor Mediated by CRISPR/Cas13a for Direct and Specific Detection of MiRNA-21

**DOI:** 10.3390/s24186138

**Published:** 2024-09-23

**Authors:** Yang Zhang, Pei Miao, Jingyuan Wang, Yan Sun, Jing Zhang, Bin Wang, Mei Yan

**Affiliations:** School of Chemistry and Chemical Engineering, University of Jinan, Jinan 250022, China; chm_zhangyang@stu.ujn.edu.cn (Y.Z.); chm-miaopei@stu.ujn.edu.cn (P.M.); faya0907@stu.ujn.edu.cn (J.W.); sunyanya@stu.ujn.edu.cn (Y.S.); zhangjingdadi@126.com (J.Z.)

**Keywords:** CRISPR/Cas13a, photoelectrochemical biosensor, miRNA-21 detection, nucleic acid signal amplification, direct recognition

## Abstract

Direct detection of miRNA is currently limited by the complex amplification and reverse transcription processes of existing methods, leading to low sensitivity and high operational demands. Herein, we developed a CRISPR/Cas13a-mediated photoelectrochemical (PEC) biosensing platform for direct and sensitive detection of miRNA-21. The direct and specific recognition of target miRNA-21 by crRNA-21 eliminates the need for pre-amplification and reverse transcription of miRNA-21, thereby preventing signal distortion and enhancing the sensitivity and precision of target detection. When crRNA-21 binds to miRNA-21, it activates the trans-cleavage activity of CRISPR/Cas13a, leading to the non-specific cleavage of biotin-modified DNA with uracil bases (biotin-rU-DNA). This cleavage prevents the biotin-rU-DNA from being immobilized on the electrode surface. As a result, streptavidin cannot attach to the electrode via specific biotin binding, reducing spatial resistance and causing a positively correlated increase in the photocurrent response. This Cas-PEC biosensor has good analytical capabilities, linear responses between 10 fM and 10 nM, a minimum detection limit of 9 fM, and an excellent recovery rate in the analysis of real human serum samples. This work presented an innovative solution for detecting other biomarkers in bioanalysis and clinical diagnostics.

## 1. Introduction

MicroRNAs (miRNAs), which comprise approximately 25 ribonucleotides, are short, non-coding, single-stranded RNA molecules that occupy a pivotal position in orchestrating diverse biological processes [1,2,3]. Recent studies have shown that miRNA mutations were closely associated with changes in the human immune system and contributed to the pathogenesis of various malignant cancers [4,5]. Among these miRNAs, miRNA-21 stands out as one of the most important cancer biomarkers and is expressed in a wide range of cancer cells [6,7,8,9]. The efficient detection of miRNA-21 is important for the early diagnosis and clinical prognosis of breast cancer. However, due to its short length, high sequence homology, low intracellular content, and unstable nature, sensitive detection of miRNA-21 poses a challenge for the scientific community. In recent years, various methods such as fluorescent labeling [10,11], colorimetric methods [12,13], surface-enhanced Raman spectroscopy [14], electrochemical labeling [15], and photoelectrochemical methods have been explored for high-sensitivity detection [16,17]. Photoelectrochemical (PEC) methodology stands apart by amalgamating the strengths of both optical and electrochemical techniques [18,19,20]. By integrating photoexcitation with electrochemical detection, it effectively reduces background signals, enhances sensitivity, and improves the stability and reliability of the system, outperforming other methods [21,22]. However, the current PEC method for miRNA detection largely relies on the reverse transcription and pre-amplification of nucleic acids, which increases the complexity of the operation [23,24]. On the one hand, processes for reverse transcription and pre-amplification of nucleic acids require a variety of enzymes, which are stored and used demandingly and are highly susceptible to environmental contamination [25]. Moreover, base-pairing errors may occur during both processes, resulting in distorted signals and false positives [26,27]. At the same time, reports have found that signal enhancement is often accompanied by a decrease in high detection sensitivity. Consequently, the development of easy-to-use signal amplification strategies for direct and specific recognition of target RNAs has emerged as a highly efficacious approach [28].

Cas13a, a member of the Type VI-a CRISPR-Cas family, functions as an RNA-guided RNA ribonuclease that non-specifically cleaves (*trans*-cleavage) nucleic acid sequences present in the system in the presence of the target miRNA. The recognition process is single nucleic acid recognition without the need for pre-amplification of the miRNAs, which greatly simplifies the process of operation and provides a novel sensing strategy for sensor signal amplification [29,30,31]. Compared to the CRISPR/Cas12a system, which only recognizes DNA targets, the CRISPR/Cas13a system can directly identify miRNA targets, avoiding signal distortion caused by reverse transcription. Additionally, the CRISPR/Cas13a system has a stronger trans-cleavage capability than CRISPR/Cas12a, significantly increasing the sensitivity and specificity of miRNA detection, with great potential for RNA detection [32,33,34]. Additionally, the presence of programmable crRNAs in the CRISPR/Cas protein system enables genetic programming for precise recognition of various miRNAs, avoiding the complex process of nucleic acid sequence design [35,36]. Apart from simple and efficient signal amplification strategies, photoelectrically active materials play a vital role in the bioanalytical performance of sensors. Recent reports have highlighted the excellent performance of the CRISPR/Cas13a protein system in photoelectrochemical sensors [37]. This sensitivity presents an opportunity to explore photoelectrically active materials that are easier to synthesize and more cost-effective. Titanium Dioxide nanorods (TiO_2_ NRs) with a controllably large specific surface area and fast charge transfer have been successfully utilized in PEC biosensing [38,39]. The TiO_2_ NRs were branched to further expand the loading area of the materials and were combined with the narrow bandgap metal sulfide semiconductor Cadmium Sulfide (CdS) to form heterojunctions [40]. This significantly broadens the visible light absorption spectrum of photoelectric materials, enhancing the utilization efficiency of visible light. It also increases the charge carrier separation rate, providing an adequate substrate signal for RNA detection, thereby facilitating more accurate and reliable analysis.

**Scheme 1:**
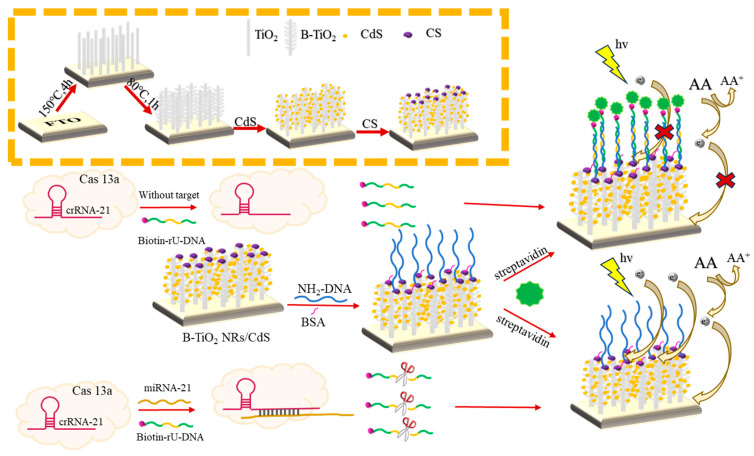
Schematic of the PEC biosensor detection of miRNA−21.

Herein, a CRISPR/Cas13a-mediated TiO_2_-based Cas-PEC biosensor was constructed for direct and sensitive analysis of miRNA-21. The cleavage activity of the Cas13a protein has been definitively confirmed using polyacrylamide gel electrophoresis. As shown in Scheme 1, the B-TiO_2_ NRs/CdS heterostructures were meticulously crafted by decorating the surface of branched TiO_2_ nanorods (B-TiO_2_ NRs) with CdS. This innovative approach combines the distinct advantages of both materials, yielding a composite with enhanced properties and expanded functionalities. The entire system was anchored by chitosan (CS) incubation, and the carboxyl groups were activated using glutaraldehyde. Subsequently, amino-modified capture DNA (NH_2_-DNA) was assembled onto B-TiO_2_ NRs/CdS/FTO. Bovine serum albumin (BSA) was used to seal inactive sites and weaken non-specific adsorption. Biotin-modified DNA probes, specifically designed with multiple ribouridine nucleotides (rUs) interspersed in their central region, were referred to as biotin-rU-DNA and used as substrates for non-specific cleavage by the CRISPR/Cas13a protein system. In the absence of miRNA-21, the unperturbed biotin-rU-DNA probe was anchored by specific pairing with NH_2_-DNA, and subsequently, biotin captured the streptavidin added to the system. In this process, the large spatial resistance caused by the size of streptavidin hinders the generation of photogenerated electrons. This also reduces the transfer of electrons from ascorbic acid (AA) to the electrodes, leading to a weakened photocurrent signal. In the existence of miRNA-21, the CRISPR/Cas13a protein system’s crRNA was specifically identified and hybridized with the target miRNA-21, triggering the system’s trans-nonspecific cleavage activity. Consequently, the biotin-rU-DNA was cleaved, preventing streptavidin from anchoring to the electrodes, thereby enhancing photoelectric signals. Notably, streptavidin’s inability to specifically interact with NH_2_-DNA mitigates false signals. This versatile Cas-PEC biosensor, through genetic customization of crRNAs, can be expanded for ultrasensitive detection of diverse miRNAs, opening new horizons for early cancer diagnosis and treatment.

## 2. Materials and Methods

Detailed information regarding all materials, reagents, and instruments used in this study is provided in the Supplementary Materials.

### 2.1. Preparation of Branched-TiO_2_ Nanorods (B-TiO_2_ NRs) Electrodes

TiO_2_ NRs were prepared using an optimized hydrothermal synthesis technique. The process begins with pre-treatment of the FTO glass substrate, followed by shaking in acetone, ethanol, and ultrapure water for 30 min prior to surface treatment. An amount of 10 mL of hydrochloric acid was mixed with 10 mL of pure water, and 400 μL of TiO_2_ was added to the solution. The mixture was then stirred vigorously for about 30 min. The solution was placed in a polytetrafluoroethylene high-pressure vessel, heated to 150 °C, and maintained at this temperature for 4 h to prepare TiO_2_ NRs. After cooling the FTO substrate to room temperature, it was rinsed with ultrapure water. Branch structures were further grown on the TiO_2_ nanorods using a hydrothermal method. A mixture of 0.25 mL TiCl_3_ and 20 mL ultrapure water was prepared in a beaker and stirred for 30 min to ensure uniform mixing.

The TiO_2_ nanorods prepared in the previous step were then placed in the beaker and heated at 80 °C for 1 h in a constant temperature device. After the reaction, the electrode was cooled to room temperature, cleaned with ultrapure water and ethanol, and dried in air. The branched TiO_2_ nanorod (B-TiO_2_ NR) electrode was obtained for later use.

### 2.2. Preparation of B-TiO_2_ NRs/CdS Electrode

The B-TiO_2_ NR electrode on FTO was immersed in a 0.1 M Cd (NO_3_)_2_ methanolic solution for 2 min. The electrode was then immersed in a 1:1 (*v*/*v*) methanolic solution of 0.1 M Na_2_S and ultrapure water for 2 min. This process was repeated five times. Afterward, the electrode was washed with ultrapure water to obtain the B-TiO_2_ NRs/CdS electrode.

### 2.3. Construction of PEC Biosensing Platform and Detection of miRNA-21

The B-TiO_2_ NR/CdS electrode (surface area: 0.28 cm^2^) was incubated with 30 μL of 1% (*w*/*v*) chitosan (CS) for 1 h, followed by the dropwise addition of 30 μL of 5% glutaraldehyde solution for 1 h, used as a fixation ligand for aminomethyl-modified NH_2_-DNA. An amount of 20 μL of 3 μM tris(2-carboxyethyl)-phosphine (TCEP) was pre-treated with 0.6 μL of 10 mM NH_2_-DNA for 1 h and then applied to the B-TiO_2_ NRs/CdS electrode. The electrode was incubated for 12 h at 4 °C in the dark, followed by three PBS buffer (pH 7.4) rinses. The electrode was then dried under nitrogen flow at room temperature. A total of 20 μL of 3% bovine serum protein solution was added to the electrode, incubated for 60 min to remove non-specifically adsorbed material, and then rinsed with 0.1 M PBS buffer (pH 7.4), yielding a BSA/NH_2_-DNA/B-TiO_2_NRs/CdS/FTO electrode. To detect miRNA-21, varying concentrations of miRNA were combined with 20 μL of a CRISPR/Cas13a system containing 25 nM Cas13a protein, 15 nM crRNA, and 200 nM biotin-rU-DNA in a buffer solution (10 mM Tris HCl, 50 mM NaCl, 1.5 mM MgCl_2_, pH 8.3). The mixture was incubated at 37 °C for 60 min to ensure thorough interaction and cleavage. The biosensing platform was completed by adding 0.5 μg/mL streptavidin to the BSA/NH_2_-DNA/B-TiO_2_ NRs/CdS/FTO electrode and incubating in the dark for 20 min. The electrode was rinsed with 0.1 M PBS buffer to clean and prepare it for further analysis.

### 2.4. Preparation of Human Serum Samples Spiked with miRNA-21

Human serum samples were obtained by centrifuging whole blood at 10,000 rpm for 10 min and then spiked with varying concentrations of miRNA-21, ranging from 10 fM to 1 nM.

## 3. Results and Discussion

### 3.1. Characterization of Heterostructures B-TiO_2_ NRs/CdS

**Figure 1:**
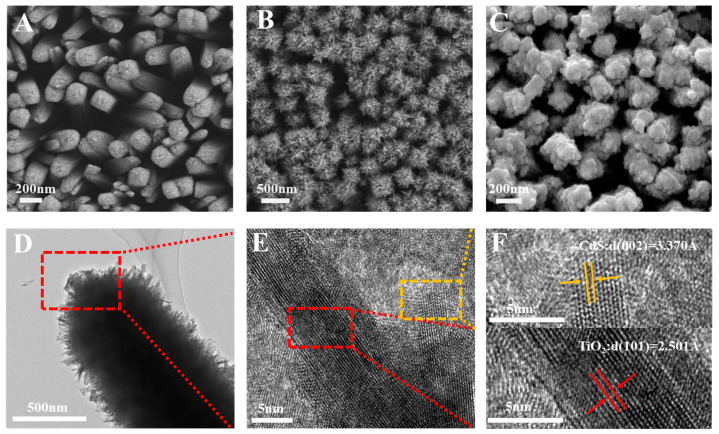
SEM images of (**A**) TiO_2_ NRs, (**B**) B-TiO_2_ NRs, and (**C**) B-TiO_2_ NRs/CdS; (**D**) TEM image of B-TiO_2_ NRs/CdS; (**E**,**F**) HR-TEM images of B-TiO_2_ NRs/CdS.

The morphology and microstructure of the TiO_2_ NRs, B-TiO_2_ NRs, and B-TiO_2_ NRs/CdS were characterized using SEM. As depicted in Figure 1A, vertically aligned TiO_2_ nanorods (NRs) were observed on the electrode surface, exhibiting a uniform height and sharply defined contours, indicative of their impeccable organization. Figure 1B shows that a dense proliferation of needle-like structures on the TiO_2_ NRs’ surface results in an enlarged diameter of the B-TiO_2_ NRs due to the inclusion of branched structures. This enlargement offered an extensive surface area poised for the subsequent loading of CdS. Following the ion exchange process, the surface became fully veiled by CdS nanoparticles, as evident in Figure 1C. The surface morphology of TiO_2_ NRs/CdS composites was meticulously analyzed using TEM (Figure 1D) and HRTEM (Figure 1E,F). Within the B-TiO_2_ NRs/CdS heterojunction, uniform CdS was decorated on the needle-like surface of B-TiO_2_ NRs, showcasing a remarkable structural integration. The precisely measured lattice fringe spacing of 0.250 nm observed within this configuration corresponds seamlessly to the (101) crystalline plane of TiO_2_, whereas the 0.370 nm lattice fringe spacing was definitively attributed to the (102) crystalline plane of CdS. The precise alignment and tight binding between these two components emphasized the robustness and consistency of the B-TiO_2_ NRs/CdS heterojunction, thus further defining the successful preparation of the B-TiO_2_ NRs/CdS composites.

**Figure 2:**
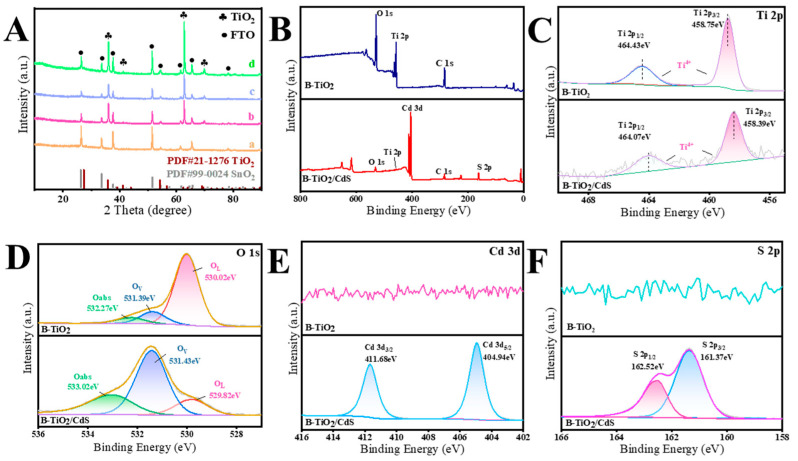
(**A**) XRD patterns of (a) FTO, (b) TiO_2_ NRs, (c) B-TiO_2_ NRs, and (d) B-TiO_2_ NRs/CdS; (**B**) XPS survey spectrum of B-TiO_2_ NRs/CdS; XPS spectra of (**C**) O 1s of B-TiO_2_ and B-TiO_2_/CdS. (**D**) Ti 2p of B-TiO_2_ and B-TiO_2_/CdS; (**E**) Cd 3d of B-TiO_2_ and B-TiO_2_/CdS; (**F**) S 2p of B-TiO_2_ and B-TiO_2_/CdS.

Further exploration of the successful preparation of the materials B-TiO2 NRs and B-TiO_2_ NRs/CdS via meticulous X-ray diffraction (XRD) mapping was conducted. Distinctive diffraction peaks observed at 26.58°, 33.86°, 37.94°, 51.75°, 54.74°, 61.86°, 65.95°, and 78.68° were characteristic markers of SnO_2_ (Figure 2A, curve a) present on the FTO substrate. Notably, the XRD image of B-TiO_2_ NRs (Figure 2A, curve c) exhibited a remarkable congruence with that of TiO_2_ NRs (Figure 2B, curve b), suggesting that the introduction of needle-like branching did not change the crystalline structure of the TiO_2_ NRs. In the XRD pattern of B-TiO_2_ NRs, a distinct set of peak intensities at 36.08°, 41.22°, 62.7°, and 69.01° corresponded precisely to the rutile TiO_2_ NRs’ (101), (111), (002), and (301) crystal planes (JCPDS.21-1276), confirming the existence of the TiO_2_ rutile phase. However, upon CdS loading, while a noticeable color alteration was observed in the material images (Figure S1), the low concentration of CdS (less than 5%) did not yield discernible CdS-related diffraction peaks in the XRD analysis (Figure 2A, curve d). Nonetheless, ICP/MS analyses conducted on the materials (Table S1) corroborated the presence of CdS on the material surfaces.

The elemental composition and surface chemical state of B-TiO_2_ NRs/CdS were investigated using XPS, demonstrating the successful preparation of B-TiO_2_ NRs/CdS composites. The XPS spectra of the B-TiO_2_ NRs/CdS composite unambiguously evidenced the coexistence of four pivotal elements: Ti; O; Cd; and S, as depicted in Figure 2B. For B-TiO_2_, the high-resolution XPS spectra of Ti _3d_ revealed distinct peaks at 464.43 eV (Ti 2p_1/2_) and 458.75 eV (Ti 2p_3/2_), definitively confirming the presence of Ti^4+^ ions within the lattice (Figure 2C). Additionally, the O 1s spectra were deconvoluted into three components: lattice oxygen (O_L_) at 530.02 eV; oxygen vacancies (O_V_) at 531.19 eV; and adsorbed oxygen (O_abs_) at 532.27 eV, providing insights into the oxygen environment within the TiO_2_ lattice (Figure 2D). Upon integration with CdS, notable shifts in the binding energies of Ti 2p and O 1s were observed. Specifically, the characteristic peaks of Ti 2p associated with Ti4+ shifted toward lower binding energies, whereas the O 1s peaks shifted toward higher binding energies. Meanwhile, the Cd 3d orbitals exhibited characteristic peaks at 404.94 eV (Cd 3d_5/2_) and 411.68 eV (Cd 3d_3/2_) in Figure 2E, conclusively verifying the incorporation of Cd^2+^ ions into the composite. These findings not only validate the successful synthesis of the B-TiO_2_ NRs/CdS composites but also provide valuable insights into their elemental composition and surface chemistry. In addition, S 2p_3/2_ and S 2p_1/2_ were responsible for the two characteristic peaks observed at 161.37 and 162.52 eV, suggesting the presence of S^2−^ within CdS (Figure 2F). The XPS results indicate that TiO_2_ and CdS coexist in B-TiO_2_ NRs/CdS composites.

Utilizing ultraviolet-visible diffuse reflectance spectroscopy, a rigorous investigation was conducted to delve into the optical properties of B-TiO_2_ NRs and their composite material with CdS, denoted as B-TiO_2_ NRs/CdS. As shown in Figure S2B, the B-TiO_2_ NRs exhibit significant absorption near 410 nm (curve a), indicating poor utilization in the visible region. After loading CdS, the absorption edge extended to around 520 nm, showing that the B-TiO_2_ NRs/CdS heterojunction enhanced absorption in the visible light range and broadened its spectral response. This led to the generation of more electron–hole pairs, an increase in photogenerated carriers and separation efficiency, and ultimately improved overall optical performance. The UV-visible diffuse reflectance curves of B-TiO_2_ were obtained by substituting Equation (1) [41]:

(1) $$\lambda =1240/E_{g},$$

*λ*: wavelength of the absorption edge; *E_g_*: bandgap energy. The estimated band gap energy of B-TiO_2_ was approximately 3.02 eV (Figure S2C), aligning well with the characteristic band gap of rutile-phase TiO_2_, confirming its compositional and structural consistency. In addition, the valence band position (E_VB_, XPS) of B-TiO_2_ could be obtained using XPS valence band spectra. After plotting the XPS VB spectra, a tangent line was made to obtain the B-TiO_2_ intersection, which corresponded to EVB, XPS, at 2.82 eV (Figure S2D). According to Equation (2), the E_VB_ at the vacuum energy level was calculated as the EVB of the corresponding standard hydrogen electrode (E_VB_, *NHE*) [42].

(2) $$E_{VB},NHE=\varphi +E_{VB},XPS-4.44,$$

*φ*: power function of the XPS instrument (4.2 eV). The E_VB_ of the prepared B-TiO_2_ could be obtained with an *NHE* of 2.58 eV. The CB and VB are similarly in accordance with Equation (3):

(3) $$E_{CB}=E_{VB}-E_{g}$$

Based on the above calculations, the energy band value of B-TiO_2_ was −0.44 eV, and the complete energy band structure of B-TiO_2_ was obtained. The energy band structure of CdS was also obtained according to the literature, which was used to derive the mechanistic diagram of the charge transfer of the system (Figure S2A) [43].

### 3.2. Feasibility of Cas13a-Mediated Cleavage

**Figure 3:**
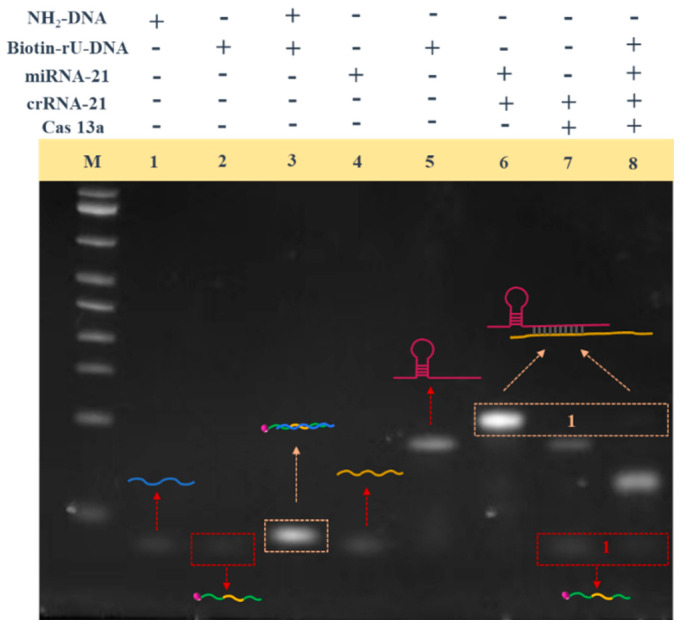
PAGE analysis of the cleavage about CRISPR/Cas13a, (lane 1) NH_2_−DNA, (lane 2) Biotin-rU−DNA, (lane 3) NH_2_−DNA + Biotin−rU−DNA, (lane 4) miRNA−21, (lane 5) crRNA−21, (lane 6) crRNA + miRNA−21, (lane 7) Cas13a + crRNA + Biotin−rU−DNA, (lane 8) Cas13a + miRNA−21 + crRNA + Biotin−rU−DNA.

As depicted in Figure 3, the efficacy of Cas13a in cleaving substrate DNA strands was thoroughly analyzed through the application of the 12% polyacrylamide gel electrophoresis (PAGE) technique, providing a clear visual representation of its cutting proficiency. After the addition of NH_2_-DNA (lane 1) and Biotin-rU-DNA (lane 2), distinct bands were observed, indicating successful migration. Notably, the crisp bands in lane 3 signify the formation of NH_2_-DNA/Biotin-rU-DNA complexes, demonstrating their spontaneous interaction to yield a double-stranded configuration. Subsequently, the introduction of miRNA-21 (lane 4) and crRNA-21 (lane 5) individually resulted in the appearance of bands at their respective positions. Intriguingly, a composite band emerged in lane 6 when both miRNA-21 and crRNA-21 were combined, suggesting that crRNA-21 effectively recognizes miRNA-21. Further experiments involving the sequential addition of Cas13a protein, Biotin-rU-DNA, and crRNA (lane 7) showed unchanged bands, indicating that Cas13a protein and crRNA alone did not initiate a cleavage response. However, upon inclusion of miRNA-21 (lane 8), a notable weakening of the miRNA-21/crRNA-21 complex bands (yellow label 1) occurred, evincing the assembly of the Cas13a-crRNA-miRNA-21 ternary complex within the system. Concurrently, a decrease in the intensity of Biotin-rU-DNA bands (red label 1) confirmed the successful activation of the Cas13a system and its efficacy in cleaving Biotin-rU-DNA.

### 3.3. Feasibility Study of PEC Biosensors

The construction process of the PEC sensing structure was monitored and evaluated through a combined approach utilizing photocurrent response analysis and electrochemical impedance spectroscopy (EIS). The EIS spectrum distinctly comprises two components: a high-frequency semicircular segment indicative of electron transfer limitations and a low-frequency linear segment representative of diffusion constraints. Notably, the diameter of this semicircular portion bears a direct correlation with the charge transfer resistance (R_et_). The B-TiO_2_ NRs/CdS/FTO electrode had the smallest value of R_et_, which became larger after the introduction of NH_2_-DNA and BSA (curve b) to the prepared substrate electrode and continued to increase after the introduction of biotin-rU-DNA (curve c). The R_et_ value reached its maximum after the introduction of streptavidin. This indicated that the rate of electron transfer was hindered due to the spatial site resistance of streptavidin. Changes in EIS data could demonstrate the successful construction of biosensors.

**Figure 4:**
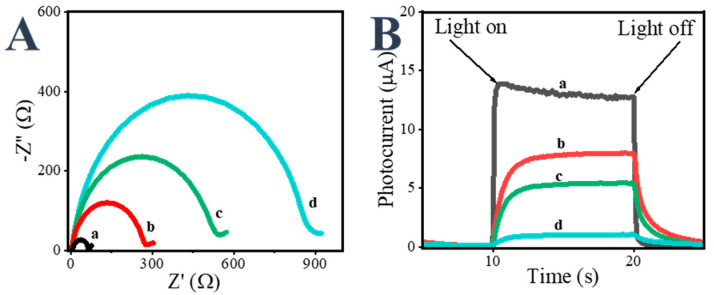
(**A**) The obtained Nyquist plots recorded in a 5 mM solution of [Fe(CN)_6_]^3−^/^4−^ with a 0.1 M solution of KCl and (**B**) photocurrents observed for various electrodes in buffer (0.1 M PBS, pH = 7.4) supplemented with AA (0.1 M) at an applied potential of 0 V: (a) B−TiO_2_ NRs/CdS; (b) BSA/NH_2_-DNA/CS/B−TiO_2_ NRs/CdS; (c) biotin−rU−DNA/BSA/NH_2_-DNA/CS/B−TiO_2_ NRs/CdS; (d) streptavidin/biotin−rU−DNA/BSA/NH_2_-DNA/CS/B−TiO_2_ NRs/CdS.

Furthermore, the step photocurrent was recorded to validate the construction process of the PEC sensor (Figure 4B). Initially, the B-TiO_2_ NRs/CdS composite exhibited a photocurrent value of approximately 13.6µA (curve a). This photocurrent underwent a decrease upon the integration of BSA and NH_2_-DNA (curve b). Subsequently, the introduction of biotin-rU-DNA probes that hybridized with NH_2_-DNA, resulting in the formation of a biotin-rU-DNA/NH_2_-DNA double-stranded structure, further diminished the photocurrent (curve c). Ultimately, the binding of streptavidin to the biotin-rU-DNA probes results in the creation of a substantial spatial obstruction. Consequently, this obstruction significantly impeded electron transfer at the electrode interface, leading to a pronounced decrease in the photocurrent, as depicted in curve d. This gradual decline in photocurrent mirrors an increasing trend in the R_et_ value, offering compelling evidence for the successful immobilization of biomolecules onto the electrode surface and the completion of the PEC biosensor assembly.

### 3.4. Optimizing Experimental Conditions

**Figure 5:**
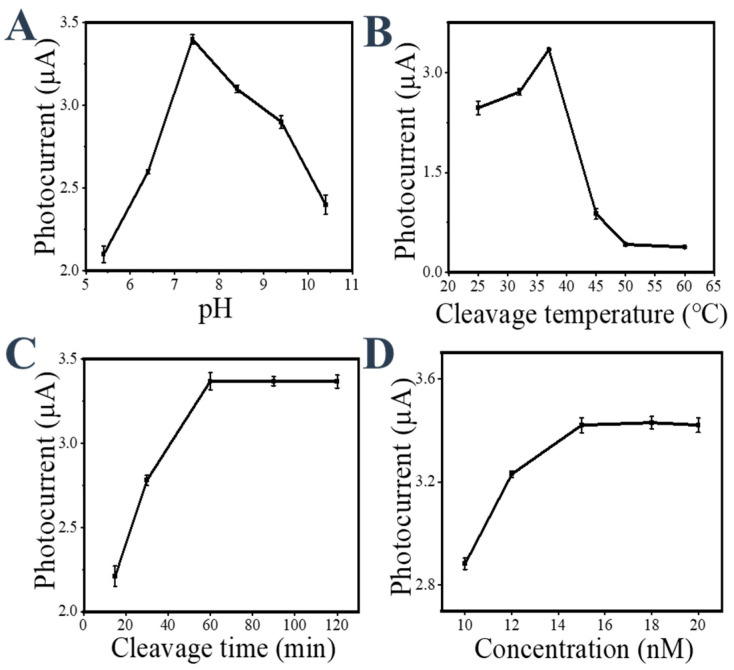
Optimization of the involved parameters: (**A**) Temperature of cleavage; (**B**) The cleavage time of the CRISPR/Cas13a complex; (**C**) Influence of pH (of PBS) on photocurrent intensity; (**D**) The concentration of crRNA-21. The experiments were carried out in the presence of 10 pM miRNA-21. The error bars present data from three independent tests.

To ensure optimal analytical performance, a thorough investigation was conducted into various crucial parameters, encompassing the pH of the assay solution as well as the cutting time and incubation temperature of the CRISPR/Cas13a system [37]. As depicted in Figure 5A, the pH of the assay solution was investigated, and the photocurrent signal varied with the pH. With the pH increasing from 5.3, the photocurrent also increased, and the photocurrent reached its highest point when the pH reached 7.4 and the photocurrent progressively decreased with further increase in pH, which indicated that a pH of 7.4 was the optimal pH value for the assay solution. Subsequently, the cutting time was assessed, as shown in Figure 5B. The photoelectric signal showed a gradual increase as more biotin-rU-DNA probes underwent cutting as the cutting time increased. The photoelectric signal reached the plateau after 60 min, and the cutting reaction was saturated. Thus, a cutting time of 60 min was determined to be the most efficient cutting time. The investigation turned to the temperature of the CRISPR/Cas13a complex’s cutting reaction, as illustrated in Figure 5C. As the reaction temperature rose, the cleavage activity of Cas13a progressively intensified, with the photocurrent signal peaking at 37 °C. However, as the temperature continued to elevate, the photocurrent signal declined and eventually plateaued. This decrease could be attributed to the detrimental effects of elevated temperatures on the cleavage activity of the Cas13a protein, leading to its inactivation. As a result, 37 °C has been established as the ideal cleavage temperature for the CRISPR/Cas13a complex, guaranteeing peak performance and robust stability for the biosensor. Finally, as shown in Figure 5D, the effect of crRNA-21 concentration on the photocurrent signal was studied, revealing that the signal varied with changes in crRNA-21 concentration. As the crRNA-21 concentration increased from 10 nM, the photocurrent signal also increased, peaking at 15 nM. However, further increases in crRNA-21 concentration resulted in diminishing changes in the photocurrent. This indicates that 15 nM is the optimal crRNA-21 concentration for this system.

### 3.5. Analytical Capabilities of PEC Biosensors for miRNA-21 Assays

**Figure 6:**
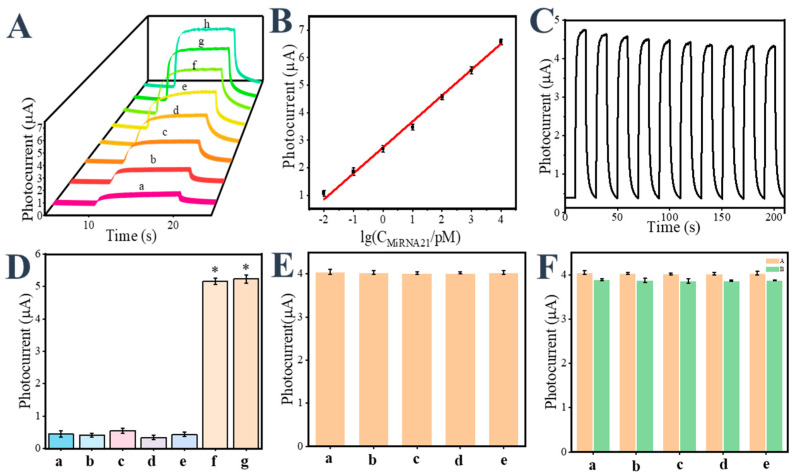
(**A**) The photocurrent responses that were obtained at various concentrations of miRNA-21 [(a–h): 0; 1 fM; 10 fM; 100 fM; 1 pM; 10 pM; 100 pM; 1 nM; and 10 nM]; (**B**) Linear regression curves of diverse miRNA-21 concentrations. The error bars present data from three independent tests; (**C**) An assessment of the sensing platform stability at 1 nM miRNA−21; (**D**) Specificity of the biosensor for miRNA assay, miRNA−21, 1 nM; the other five miRNAs, 1 nM; a mixture comprising miRNA−21 (1 nM) and the five miRNAs (each miRNA at 1 nM concentration). a: miRNA−17, b: miRNA−122, c: miRNA−155, d: miRNA−199, e: miRNA−210, f: mixture, g: miRNA−21; two-tailed Student’s *t*−test, *, *p* < 0.05 (**E**) Photocurrents of five batches of electrodes under the same test conditions at the same time; (**F**) Photocurrents of five batches of electrodes after two weeks (A: fresh, B: after two weeks). The error bars present data from three independent tests.

The photocurrent size was related to the concentration of miRNA-21, and the detection ability of the constructed biosensor was evaluated by analyzing different concentrations of miRNA-21 and plotting the corresponding i-t curves. As depicted in Figure 6A, a gradual enhancement in the photocurrent was observed with an increasing concentration of miRNA-21, ranging from 10 fM to 10 nM. Furthermore, Figure 6B reveals a pronounced linear correlation between the variation in PEC response and the logarithmic concentration of miRNA-21, adhering to Equation (4):


I = 0.6565lgC_miRNA_ + 1.8309 (R^2^ = 0.9902) (4)

C represents the miRNA concentration; I signifies the biosensor’s photocurrent signal. Based on these findings, the biosensor achieved a remarkable detection limit of 9 fM (S/N = 3). Table 1 summarizes other detection methods for miRNA-21; the PEC biosensor developed in this work expands the detection range of miRNA-21. It achieves a lower or comparable detection limit compared to other reported methods, and this highlights its high sensitivity in detecting miRNA-21.

### 3.6. The Study of Sensor’s Stability, Selectivity, and Reproducibility

Reproducibility, stability, and selectivity were the key characteristics for detecting the good or bad performance of the sensor. Initially, we delved into the stability of the biosensor, as depicted in Figure 6C. The photocurrent was tested in a PBS buffered salt solution during 10 on/off light source cycles, with the entire cycle lasting 200 s. Notably, during the initial two cycles, the photocurrent exhibited a slight downward trend, followed by minimal fluctuations in subsequent iterations, signifying commendable stability within the PEC biosensor. Subsequent to stability assessment, the biosensor’s specificity was interrogated by detecting interfering miRNAs featuring disparate sequences. As shown in Figure 6D, the photocurrent generated by 1 nM miRNA-21 exceeded the other five miRNAs, each at a concentration of 1 nM. Furthermore, when 1 nM miRNA-21 was mixed with the five other miRNAs (each at 1 nM), the resultant photocurrent showed minimal deviation from that of miRNA-21 measured individually. This observation underscored the exceptional selectivity of the developed biosensor platform for the specific detection of miRNA-21.

Five PEC biosensors from the same batch were tested with 10 pM miRNA-21 to observe their photocurrent responses (Figure 6E). The minimal variation in signal among these sensors indicated their reproducibility, with a relative standard deviation of 1.27%. To further rigorously evaluate the reproducibility, we conducted additional tests using 10 pM miRNA-21 on the PEC biosensors. As depicted in Figure 6F, to assess the long-term stability, five distinct batches of electrodes were stored under refrigerated conditions (4 °C) for two weeks. Notably, the corresponding results presented in Figure 6F demonstrate that the recorded data retained 96.03% of the performance compared to freshly fabricated PEC biosensing electrodes, conclusively validating the exceptional long-term storage stability of this biosensor.

### 3.7. Serum Sample Analysis

To demonstrate the efficacy of the PEC sensor in sensitively detecting miRNA-21 within authentic biological samples, we employed the PEC sensor for the analysis of human serum. As presented in Table 2, the recovery rates of the spiked miRNA-21 achieved remarkable values of 97.7%, 106.0%, 101.3%, 98.9%, and 96.4%, respectively. These results underscore the immense potential and practical feasibility of this platform for clinical diagnostic applications.

## 4. Conclusions

In conclusion, this study successfully fabricated a novel, highly sensitive PEC biosensor that utilized the CRISPR/Cas13a system for the direct and specific detection of miRNA-21. The CRISPR/Cas13a system, known for its exceptional trans-cleavage activity, allowed for precise recognition of the target miRNA, which then triggered trans-cleavage. This innovative recognition mechanism formed the cornerstone of the biosensor’s signal amplification design, highlighting its potential. Furthermore, the versatility of this PEC biosensor can be extended to ultra-sensitive detection of diverse miRNAs by strategically programming crRNAs. This approach simplifies traditional nucleic acid sequence design, streamlining the construction of the biosensing platform and significantly enhancing its adaptability. In conclusion, the exceptional analytical capabilities of these biosensors, particularly their ability to detect low-abundance miRNAs, position them as promising tools for transformative advancements in biomedical research and clinical diagnostics.

## Figures and Tables

**Table 1:** Comparison of various analytical methods for miRNA-21 detection.

Analytical Methods	Linear Range	Detection Limit	References
Electrochemistry Electrochemiluminescence Colorimetry Fluorescence Photo electrochemistry Photo electrochemistry Photo electrochemistry	1 pM~10 nM 0.02 pM~120 pM 100 fM~10 nM 0.05 nM~10 nM 1 fM to 5 nM 1 pM to 100 nM 10 fM~10 nM	0.26 pM 6.3 fM 36.2 fM 1 pM 1 fM 0.31 pM 9 fM	[44] [45] [46] [47] [37] [48] This work

**Table 2:** Determination of miRNA-21 recovery in human serum samples.

Sample (Number)	Quantity Added	Found	Recovery Rate (%)
1	10 fM	9.77 ± 0.29 fM	97.7
2	0.1 pM	0.106 ± 0.02 fM	106.0
3	100 pM	101.2 ± 1.11 fM	101.3
4	1 nM	0.988 ± 0.03 fM	98.9
5	10 nM	9.63 ± 0.02 fM	96.4

## Data Availability

The data supporting this article have been included in the article and Supplementary Materials.

## Supplementary Materials

The following supporting information can be downloaded at https://www.mdpi.com/article/10.3390/s24186138/s1, Figure S1: Actual photographs of the material; Figure S2: (**A**) Photo−generation electron-transfer mechanism at B−TiO_2_ NRs/CdS/FTO electrode; (**B**) UV–vis absorption for (**a**) B−TiO_2_ NRs and (**b**) B−TiO_2_ NRs/CdS; (**C**) UV−vis diffuse reflectance spectra of B-TiO_2_ were converted to obtain the corresponding (Ahν)^2^−hν maps; (**D**) VB−XPS maps of B-TiO_2_; Table S1: Molar ratio of Ti, Cd that can be obtained by ICP/MS; Table S2: Oligonucleotide sequences used in this work.
