# Supplementary material for: A Photoelectrochemical Biosensor Mediated by CRISPR/Cas13a for Direct and Specific Detection of MiRNA-21
Source: Sensors (Basel). 2024 Sep 23;24(18):6138. doi: 10.3390/s24186138 (PMC11436169; doi:10.3390/s24186138)
Supplement: Supplementary file 1 [file sensors-24-06138-s001.zip › sensors-3178158-supplementary.pdf]

Supplementary Materials

A Photoelectrochemical Biosensor Mediated by Cas13a for Direct and Specific Detection of MiRNA-21

Yang Zhang[a], Pei Miao[a], Jingyuan Wang[a], Yan Sun[a], Jing Zhang[a], Bin Wang[a*], Mei Yan[a*]

[a]*School of Chemistry and Chemical Engineering, University of Jinan, Jinan 250022, P.R. China.*

***Corresponding Authors:**

Mei Yan (chm_yanm@ujn.edu.cn);

Bin Wang (chm_wangbin@ujn.edu.cn)

Table of contents

1.Materials..S3

2.Apparatus..S4

3. Preparation of branched-TiO_2 nanorods (B-TiO_2 NRs) electrodes..........................S5

4. Preparation of B-TiO_2 NRs/CdS electrode..S6

5. Construction of PEC biosensing platform and detection of miRNA-21.................S7

6.Figure S1..S8

7.Figure S2..S9

8.Table S1..S10

9.Table S2..S11

Materials

Fluorine-doped tin oxide (FTO) glass substrate with a thickness of 2.2 mm (resistance of <15 Ω/square) was purchased from Xiamen FTO Photoelectricity Industry. Titanium trichloride ($TiCl_3$, >98%) was obtained from Sinopharm Chemical Reagent Co. Ltd. (Shanghai, China). Cadmium nitrate (Cd $(NO_3)_2 \cdot 4H_2O$) and sodium sulfide ($Na_2S \cdot 9H_2O$) were obtained from Nanjing Chemical Reagent Co., Ltd. (China). All the oligonucleotides were synthesized by Sangun Biotech (Shanghai, China). An amount of 20× phosphate buffer saline (PBS), streptavidin, ascorbic acid, NaCl, and $MgCl_2$ were purchased from Sangun Biotech (Shanghai, China). K_3[Fe $(CN)_6$] $FeCl_3$ was bought from Beijing Reagent Company. Lipoteichoic bucktails (Lbu) Cas13a protein was supplied by BioLife Sciences Ltd. (Guangzhou, China). The solvent in each step was deionized water (DI water, 18 MΩ/cm) from a Milli-Q water purification system.

Apparatus

The scanning electron microscopy (SEM) images were carried out by an S-4800 scanning electron microscope (Hitachi Ltd., Japan). The powder X-ray diffraction (XRD) was measured on a Philips X'pert Pro X-ray diffractometer in the 2θ range from 10° to 80°, with Cu Kα radiation of 0.15418 nm (Netherlands). The UV-visible (UV-vis) absorption spectra were obtained on a UV-3600 UV-visible spectrophotometer (Shimadzu, Japan). Electrochemical impedance spectroscopy (EIS) was performed on an Autolab potentiostat/galvanostat (PGSTAT 30, Eco Chemise B.V., Utrecht, Netherlands) with a three electrodes system in 0.1 M KCl solution containing 2.0 mM $K_3[Fe (CN)6]/K_4[Fe (CN)_6]$ (1:1) mixture as a redox probe, and recorded in the frequency range of 0.01 Hz-100 kHz with an amplitude of 50 mV. PEC measurements were carried out with a homemade PEC system. A 500 W Xe lamp was used as the irradiation source with a light intensity of 400 $\mu W \cdot cm^{-2}$ estimated by a radiometer (Photoelectric Instrument Factory of Beijing Normal University). The photocurrent was recorded on a CHI 660D electrochemical workstation (Shanghai Chenhua Apparatus Corporation, China) using a three-electrode system, with 0.25 cm^2 modified FTO electrode, saturated Ag/AgCl electrode, and platinum wire as the working, reference, and counter electrode, respectively.

Preparation of branched-TiO_2 nanorods (B-TiO_2 NRs) electrodes.

TiO_2 NRs were prepared using an optimized hydrothermal synthesis technique. The process begins with pre-treatment of the FTO glass substrate, followed by shaking in acetone, ethanol, and ultrapure water for 30 minutes prior to surface treatment. An amount of 10 mL of hydrochloric acid was mixed with 10 mL of pure water, and 400 μL of TiO_2 was added to the solution. The mixture was then stirred vigorously for about 30 minutes. The solution was placed in a polytetrafluoroethylene high-pressure vessel, heated to 150 °C, and maintained at this temperature for 4 hours to prepare TiO2 NRs. After cooling the FTO substrate to room temperature, it was rinsed with ultrapure water. Branch structures were further grown on the TiO_2 nanorods using a hydrothermal method. A mixture of 0.25 mL $TiCl_3$ and 20 mL ultrapure water was prepared in a beaker and stirred for 30 minutes to ensure uniform mixing.

The TiO_2 nanorods prepared in the previous step were then placed in the beaker and heated at 80°C for 1 hour in a constant temperature device. After the reaction, the electrode was cooled to room temperature, cleaned with ultrapure water and ethanol, and dried in air. The branched TiO_2 nanorod (B-TiO_2 NR) electrode was obtained for later use.

Preparation of B-TiO_2 NRs/CdS electrode

The B-TiO_2 NR electrode on FTO was immersed in a 0.1 M Cd $(NO_3)_2$ methanolic solution for 2 minutes. The electrode was then immersed in a 1:1 (v/v) methanolic solution of 0.1 M Na_2S and ultrapure water for 2 minutes. This process was repeated five times. Afterward, the electrode was washed with ultrapure water to obtain the B-TiO_2 NRs/CdS electrode.

Construction of PEC biosensing platform and detection of miRNA-21

The B-TiO_2 NR/CdS electrode (surface area: 0.28 cm^2) was incubated with 30 μL of 1% (w/v) chitosan (CS) for 1 hour, followed by the dropwise addition of 30 μL of 5% glutaraldehyde solution for 1 hour, used as a fixation ligand for aminomethyl-modified NH_2-DNA. 20 μL of 3 μM tris(2-carboxyethyl)-phosphine (TCEP) was pre-treated with 0.6 μL of 10 mM NH_2-DNA for 1 hour, then applied to the B-TiO2 NRs/CdS electrode. The electrode was incubated for 12 hours at 4 °C in the dark, followed by three PBS buffer (pH 7.4) rinses. The electrode was then dried under nitrogen flow at room temperature. A total of 20 μL of 3% bovine serum protein solution was added to the electrode, incubated for 60 minutes to remove non-specifically adsorbed material, and then rinsed with 0.1 M PBS buffer (pH 7.4), yielding a BSA/NH_2-DNA/B-TiO_2NRs/CdS/FTO electrode. To detect miRNA-21, varying concentrations of miRNA were combined with 20 μL of a CRISPR/Cas13a system containing 25 nM Cas13a protein, 15 nM crRNA, and 200 nM biotin-rU-DNA in a buffer solution (10 mM Tris HCl, 50 mM NaCl, 1.5 mM $MgCl_2$, pH 8.3). The mixture was incubated at 37 °C for 60 minutes to ensure thorough interaction and cleavage. The biosensing platform was completed by adding 0.5 μg/mL streptavidin to the BSA/NH_2-DNA/B-TiO_2 NRs/CdS/FTO electrode and incubating it in the dark for 20 minutes. The electrode was rinsed with 0.1 M PBS buffer to clean and prepare it for further analysis.

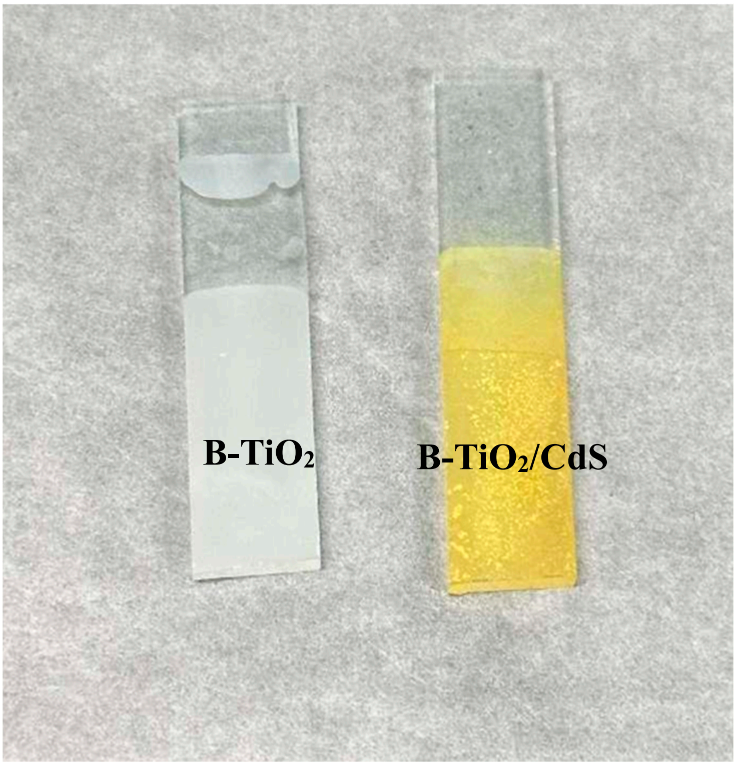

Figure S1. Actual photographs of the material.

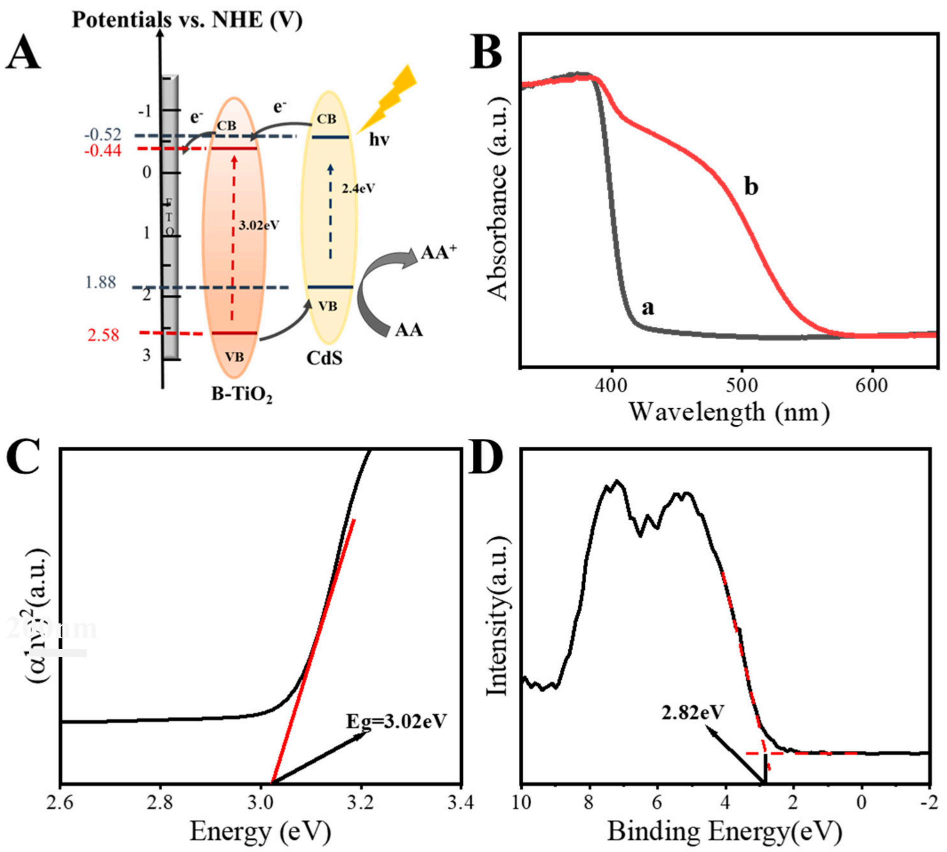

Figure S2. (A) Photo-generation electron-transfer mechanism at B-TiO_2 NRs/CdS/FTO electrode. (B) V-vis absorption for (a) B-TiO_2 NRs and (b) B-TiO_2 NRs/CdS. (C) UV-vis diffuse reflectance spectra of B-TiO_2 were converted to obtain the corresponding $(Ah\nu)^2$-hν maps. (D) VB-XPS maps of B-TiO_2.

Table S1. Molar ratio of Ti, Cd that can be obtained by ICP/MS

Test element	Sample element content w (%)	Sample element molar ratio
Ti	0.754	3.95
Cd	0.447	1

Table S2. Oligonucleotide sequences used in this work.

Name	Sequence (from 5' to 3')
miRNA-21	UAGCUUAUCAGACUGAUGUUGA
miRNA-	GACCACCCCAAAAAUGAAGGGGACUAAAACAACAUCA
21cRNA	GUCUGAUAAGCUA
	NH_2-CGCTGTCTGGTCGAAATGAG
NH_2-DNA	Biotin-CTCArUrUrUCGACCAGACAGCG
Biotin-RU-	CAAAGUGCUUACAGUGCAGGUAG
DNA	UGGAGUGUGACAAUGGUGUUUG
miRNA-17	UUAAUGCUAAUCGUGAUAGGGGU
miRNA-122	ACAGUAGUCUGCACAUUGGUUA
miRNA-155	AGCCCCUGCCCACCGCACACUG
miRNA-199	
miRNA-210	
